# Earliest giant panda false thumb suggests conflicting demands for locomotion and feeding

**DOI:** 10.1038/s41598-022-13402-y

**Published:** 2022-06-30

**Authors:** Xiaoming Wang, Denise F. Su, Nina G. Jablonski, Xueping Ji, Jay Kelley, Lawrence J. Flynn, Tao Deng

**Affiliations:** 1 Department of Vertebrate Paleontology, Natural History Museum of Los Angeles County, 900 Exposition Blvd., Los Angeles, CA 90007 USA; 2 Key Laboratory of Vertebrate Evolution and Human Origins of Chinese Academy of Sciences, Institute of Vertebrate Paleontology and Paleoanthropology, Chinese Academy of Sciences, Beijing, 100044 China; 3 Institute of Human Origins and School of Human Evolution and Social Change, Arizona State University, Tempe, AZ 85281 USA; 4 Department of Anthropology, Pennsylvania State University, University Park, PA 16802 USA; 5 Kunming Natural History Museum of Zoology, Kunming Institute of Zoology, Chinese Academy of Sciences, Kunming, 650201 China; 6 Yunnan Institute of Cultural Relics and Archaeology, 15-1, Chunmingli, Chunyuan Xiaoqu, Kunming, 650118 Yunnan China; 7 Department of Human Evolutionary Biology, Harvard University, Cambridge, MA 02138 USA

**Keywords:** Evolution, Palaeontology

## Abstract

Of the many peculiarities that enable the giant panda (*Ailuropoda melanoleuca*), a member of the order Carnivora, to adapt to life as a dedicated bamboo feeder, its extra “thumb” is arguably the most celebrated yet enigmatic. In addition to the normal five digits in the hands of most mammals, the giant panda has a greatly enlarged wrist bone, the radial sesamoid, that acts as a sixth digit, an opposable “thumb” for manipulating bamboo. We report the earliest enlarged radial sesamoid, already a functional opposable “thumb,” in the ancestral panda *Ailurarctos* from the late Miocene site of Shuitangba in Yunnan Province, China. However, since the late Miocene, the “thumb” has not enlarged further because it must be balanced with the constraints of weight bearing while walking in a plantigrade posture. This morphological adaptation in panda evolution thus reflects a dual function of the radial sesamoid for both bamboo manipulation and weight distribution. The latter constraint could be the main reason why the panda’s false thumb never evolved into a full digit. This crude “thumb” suggests that the origin of the panda’s dedicated bamboo diet goes back to as early as 6–7 Ma.

## Introduction

The false thumb of the giant panda (“panda” throughout text below unless otherwise specified) fascinated early naturalists^1–3^. In recent decades, as popularized by Gould^4,5^, it has become a celebrated case of evolutionary adaptation to independently acquire an opposable thumb-like structure when the need arose. Gould’s essay also highlights an exclusive association of this unique anatomic structure with an equally unique diet of bamboo herbivory, although a false thumb has been shown also to have evolved independently in the red panda and its distant relatives^6,7^, in tremarctine bears (either convergently or as a shared plesiomorphic trait)^8^, as well as in a distant relative of the giant panda clade^9^. In fact, the giant panda is a striking example of a highly specialized member of the bear family (Ursidae) that has become a dedicated herbivore, a rare case of a large carnivore with a short, carnivorous digestive tract^10^ that became a low-level consumer with a greatly altered gut microbiota^11^.

Despite its celebrated status, the panda’s false thumb is a small, flat structure that barely protrudes out of the palmar surface, and this relatively obscure anatomy understandably baffled early anatomists (e.g., Wood-Jones^3^). Such a relatively small and flat radial sesamoid has also been documented in fossil pandas from the late Pleistocene (ca. 102–49 Ka) Shuanghe Cave^12^. If bamboo manipulation is the main function of this feature, why did pandas not evolve a markedly more elongated radial sesamoid, one that more closely resembles a true opposable thumb for the efficient gripping of bamboo, given that mammalian sesamoids seem to be readily elongated with relatively little developmental constraint^13^? Until now, this question has not been answerable due to a lack of fossil evidence beyond late Pleistocene within the *Ailurarctos-Ailuropoda* lineage.

**Figure 1:**
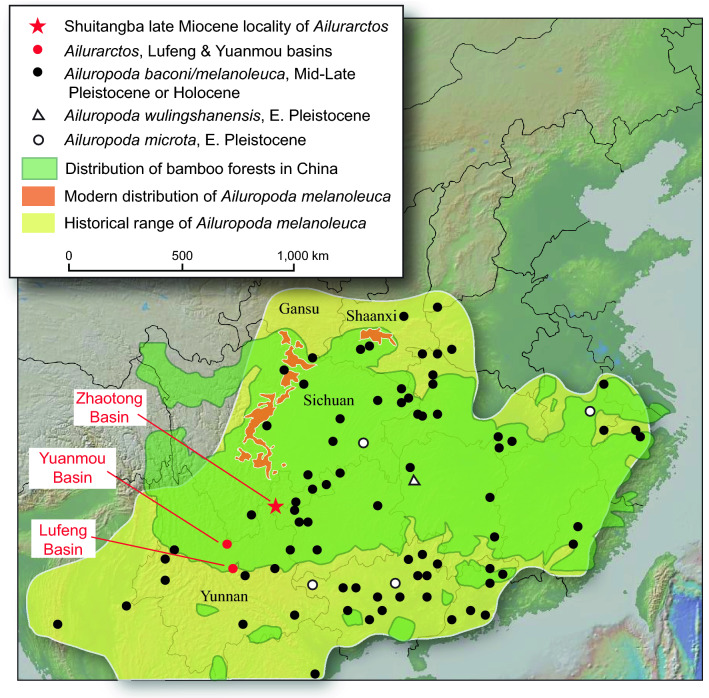
Map of southern China showing late Miocene localities with panda specimens in Yunnan (red symbols), the giant panda’s modern distribution (orange)^16^ and historical range (yellow)^16–18^, present approximate bamboo forest distribution in China (green)^19^, and Pleistocene and later fossil distribution (solid and open black circles and triangles)^16,18–24^. We eliminated the humerus record from Zhoukoudian locality 1 following Jiangzuo et al.^25^ (see discussion in online Supplementary material). Topographical map generated by GeoMapApp (version 3.6.10) under CC BY license^26^.

We report here the earliest occurrence of an enlarged radial sesamoid, as well as an isolated M2, a broken canine, and a partial humerus, all assigned to *Ailurarctos*, from Shuitangba, a late Miocene site in the Zhaotong Basin, Yunnan Province (Fig. 1). The morphology of the preserved dentition closely matches that of the stem genus *Ailurarctos* (tribe Ailuropodini) from Lufeng and Yuanmou^14,15^, the most basal panda so far known. The false thumb in *Ailurarctos* shows an intermediate morphology (see below), and thus documents, for the first time, the likely timing and steps in the evolution of bamboo feeding in pandas.

**Institutional Abbreviations**. **IVPP**, Institute of Vertebrate Paleontology and Paleoanthropology, Chinese Academy of Sciences, Beijing; **KIZ**, Kunming Institute of Zoology, Chinese Academy of Sciences, Kunming; **USNM**, National Museum of Natural History, Smithsonian Institution, Washington, D.C.; **YV**, Yunnan Institute of Cultural Relics and Archaeology, Kunming, Yunnan Province; **ZT**, Zhaotong collection, Yunnan Institute of Cultural Relics and Archaeology, Kunming, Yunnan Province.

### Systematic paleontology

Order Carnivora Bowdich, 1821.

Family Ursidae Fischer von Waldheim, 1817.

Subfamily Ailuropodinae Grevé, 1894.

Tribe Ailuropodini Grevé, 1894.

Genus *Ailurarctos* Qiu and Qi, 1989.

*Ailurarctos* cf. *A. lufengensis* Qiu and Qi, 1989.

**Referred specimens**. From Shuitangba, Zhaotong Basin, Yunnan: ZT-2015-0124, an isolated left M2 (Fig. 2A-C); ZT-2015-0056, left radial sesamoid (Figs. 3, 4, S1); ZT-2007-02-097, partial lower canine (Fig. S2); ZT-2007-62-251, distal half of left humerus (Fig. S3). See online Supplementary material for depositional context and associated fauna.

**Figure 2:**
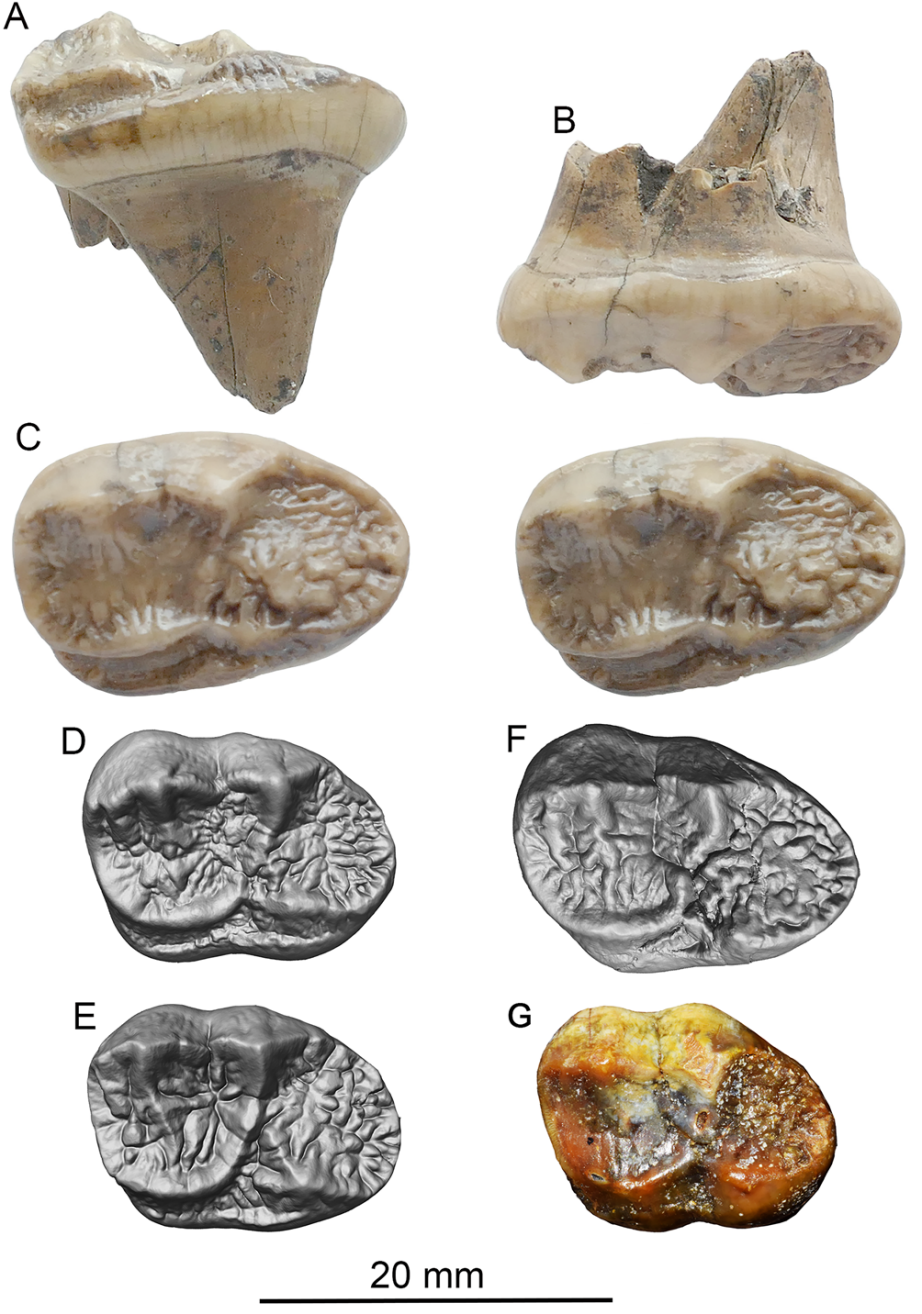
*Ailurarctos* cf. *A. lufengensis* from Zhaotong (**A**–**C**) compared to *A. lufengensis* from the type locality of Lufeng (**D**–**F**) and *A. yuanmouensis* from type locality of Yuanmou (**G**). ZT-2015–0124, (**A**) lingual, (**B**) labial, and (**C**) occlusal (stereophoto) of left M2; (**D**) right M2 (reversed), IVPP V6892.4; (**E**) left M2, IVPP V6892.5, and (**F**) left M2, IVPP V6892.6; and (**G**) left M2, YV 2509.2, *A. yuanmouensis*. Images for *A. lufengensis* and *A. yuanmouensis* are courtesy of Qigao Jiangzuo.

**Figure 3:**
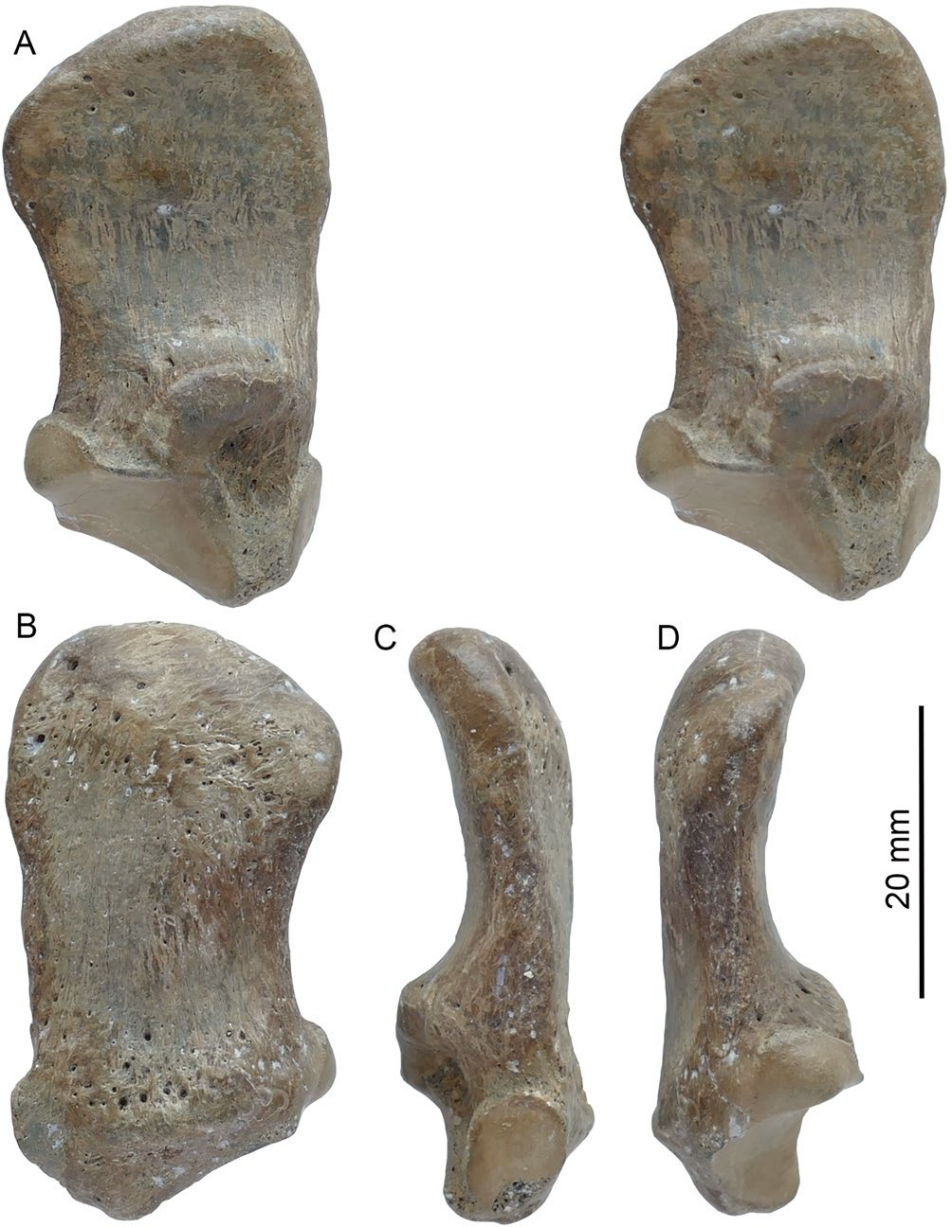
*Ailurarctos* cf. *A. lufengensis*, ZT-2015–0056, left radial sesamoid, (**A**) left lateral (in stereo), (**B**) medial, (**C**) proximal, and (**D**) distal views.

**Figure 4:**
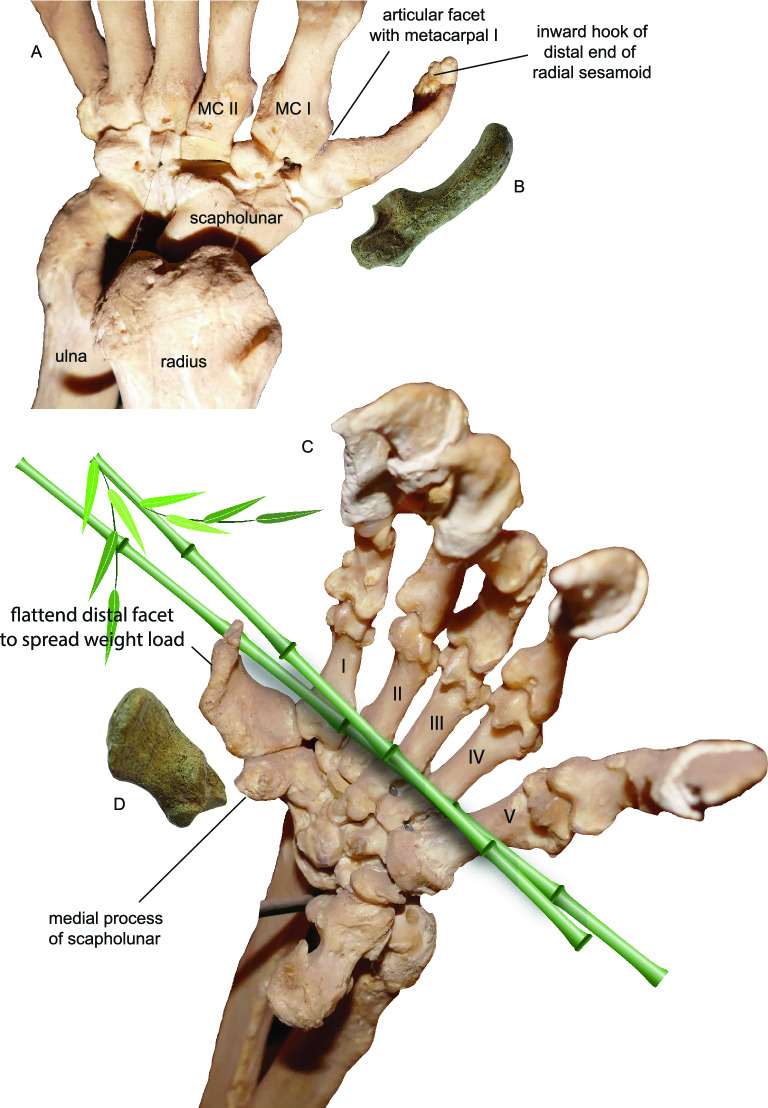
Giant panda’s false thumb. Dorsal (**A**) and ventral (**C**) views of the modern giant panda left hand, as compared with an isolated left radial sesamoid of *Ailurarctos* cf. *A. lufengensis* (**B** and **D**, ZT-2015–0056) at a similar angle and relative size. Mounted skeleton of the giant panda on display at KIZ exhibition hall, probably a zoo specimen.

**Comparison of M2, taxonomic assignment, and diet**. Three M2s of *Ailurarctos lufengensis* (Fig. 2D-F) and one of *A. yuanmouensis* (Fig. 2G) are available and offer a sense of the variation within and between species. The three M2s from Lufeng display a range of morphology: presence or absence of a lingual cingulum and reduction of buccal cingulum, and presence or absence of a clearly delineated metaconule (RMe3 in Jiangzuo et al.^27^). Of these, the Shuitangba M2 (ZT-2015-0124) has a distinct lingual cingulum but lacks an elevated metaconule. The Lufeng sample, however, consistently has a more distinct ridge on the lingual side of the preparacrista (RPa1.2 in Jiangzuo et al.^27^) not seen in ZT-2015-0124. An M2 of *A. yuanmouensis* has substantially less crenulation in its trigon, whereas its talon has achieved the level of complexity seen in those from Lufeng and Shuitangba. The overall proportion of the Shuitangba M2 is slightly more elongated than in the Lufeng sample and the elevated talon anterocentrally is not seen in the Lufeng M2s. Since M2s in *Ailuropoda* are more quadrate with greater width to length ratios (Table 1), the narrower ZT-2015-0124 appears slightly more primitive. Given the above dental comparisons, the Shuitangba M2 reliably belongs to *Ailurarctos*, in terms of both the length/width proportions and the detailed cusp morphology, and we tentatively assign it to *Ailurarctos* cf. *A. lufengensis* pending additional materials becoming available.

**Table 1:** Comparison of dental measurements (in mm).

	*Ailurarctos yuanmouensis*	*Ailurarctos lufengensis*	*Ailurarctos* cf. *A. lufengensis*	*Ailuropoda microta*	*Ailuropoda wulingshanensis*	*Ailuropoda baconi*	*Ailuropoda melanoleuca*
	Zong^15^	Qiu and Qi^14^	ZT-2015–0124	Jin et al.^20^	Jin et al.^20^	Pei^49^	Colbert and Hooijer^50^
	n = 1	n = 2	n = 1	n = 25	n = 36	n = 41	n = 19
M2 length	16.0	17.6–19.8	22.2	20.0–25.0	24.2–32.5	31.0–40.5	30.4–36.5
M2 width	12.8	14.2–14.9	14.3	16.0–20.0	19.0–26.0	23.7–30.5	24.0–28.2

Among carnivorans, ursids have the most complex molars due to their unique morphology related to hypocarnivory. Within ursids, dental patterns in ailuropodines are some of the most elaborate, with numerous, highly distinct crown cuspules^27^, advantageous for crushing tough bamboo, i.e., durophagous mastication^20^. These features are associated with a robust mandible^28^ and lateral movements of the temporomandibular joint^29^. It is evident that the dental pattern of *Ailurarctos* has reached the level of complexity of modern *Ailuropoda*, as recognized by Qiu and Qi^14^. In fact, the degree of enamel crenulation on most M2s of *Ailurarctos* is even greater than in *Ailuropoda*. If it is accepted that the robust cuspation in *Ailuropoda* is linked to a bamboo diet, dental specializations in *Ailurarctos* strongly suggest both an ancestral relationship to *Ailuropoda* as well as a diet including bamboo^20^. See additional description and comparison of other specimens in online Supplementary material.

**Description and comparison of the radial sesamoid**. The radial sesamoid, ZT-2015-0056 (Figs. 3, 4), resembles in all essential details those of *Ailuropoda* previously described^10,12,30^. Compared to those of *Indarctos arctoides*, a possible stem ailuropodine in the late Miocene of Spain, the *Ailurarctos* radial sesamoid is considerably larger, relatively wider, and more hooked (see relative size of radial sesamoid to metacarpal I of *I. arctoides* in Fig. 8 of Abella et al.^9^), although we lack knowledge of *Ailurarctos* metacarpals and Abella et al. did not publish lengths of metacarpal I. The proximal articulating facets are also much larger with a more concave facet for the scapholunar in *Ailurarctos*, while the distal end of the *I. arctoides* radial sesamoid still preserves a possible cartilaginous tip (see Fig. 3 of Abella et al.^9^), which is also present in some extant *Ailurus*^7^ but presumably absent in *Ailurarctos* (lacking a distinct rim seen in *Indarctos*). The radial sesamoid of *Ailurarctos* is slightly larger than those of modern pandas, by 8% if compared to the maximum length of the largest radial sesamoid of living panda measured by Li and others^31^, but relative to body size (using M2 length as a proxy), it is significantly larger than its modern counterparts (Table 2). It is gently convex on the external (approximately ventral) surface and concave on the internal surface (orientation assuming a plantigrade posture). At the proximal end, a large, elongate, concave facet (16 mm in maximum longitudinal dimension) articulates with the medial process of the scapholunar, whereas a much smaller, oval-shaped, flat facet (7 × 9 mm) articulates with the medial face of the first metacarpal. The distal end thickens slightly and bends toward the palm, as if to oppose to the fingers.

**Table 2:** Measurements of radial sesamoids (in mm).

	*Ailurarctos* cf. *A. lufengensis*	*Ailuropoda melanoleuca* (Pleistocene)	*Ailuropoda melanoleuca* (modern)
	ZT-2015–0056	19SZD-12	Li et al.^31^
Greatest proximal distal length	42.0	34.0	31–39
Least width at mid shaft	18.3	23.5	13–17
Least thickness at mid shaft	7.6		6–8
Radial sesamoid index	1.89	0.92	0.84–1.28

Radial sesamoid index is bone length/M2 length (the latter from Table 1 as a proxy for body size). This shows the size of the false thumb relative to body size. Radial sesamoid data for a late Pleistocene *Ailuropoda melanoleuca* from Shuanghe Cave is from Wang et al.^12^; average M2 length for 12 individuals from this cave (36.87 mm) kindly provided by Qigao Jiangzuo. Note that for *Ailuropoda melanoleuca*, we used the radial sesamoid length range in Li and others^31^ divided by M2 length range from Colbert and Hooijer^50^, and the resulting radial sesamoid index range reflects the likely values that would exceed actual ratios if derived from individual specimens.

**Figure 5:**
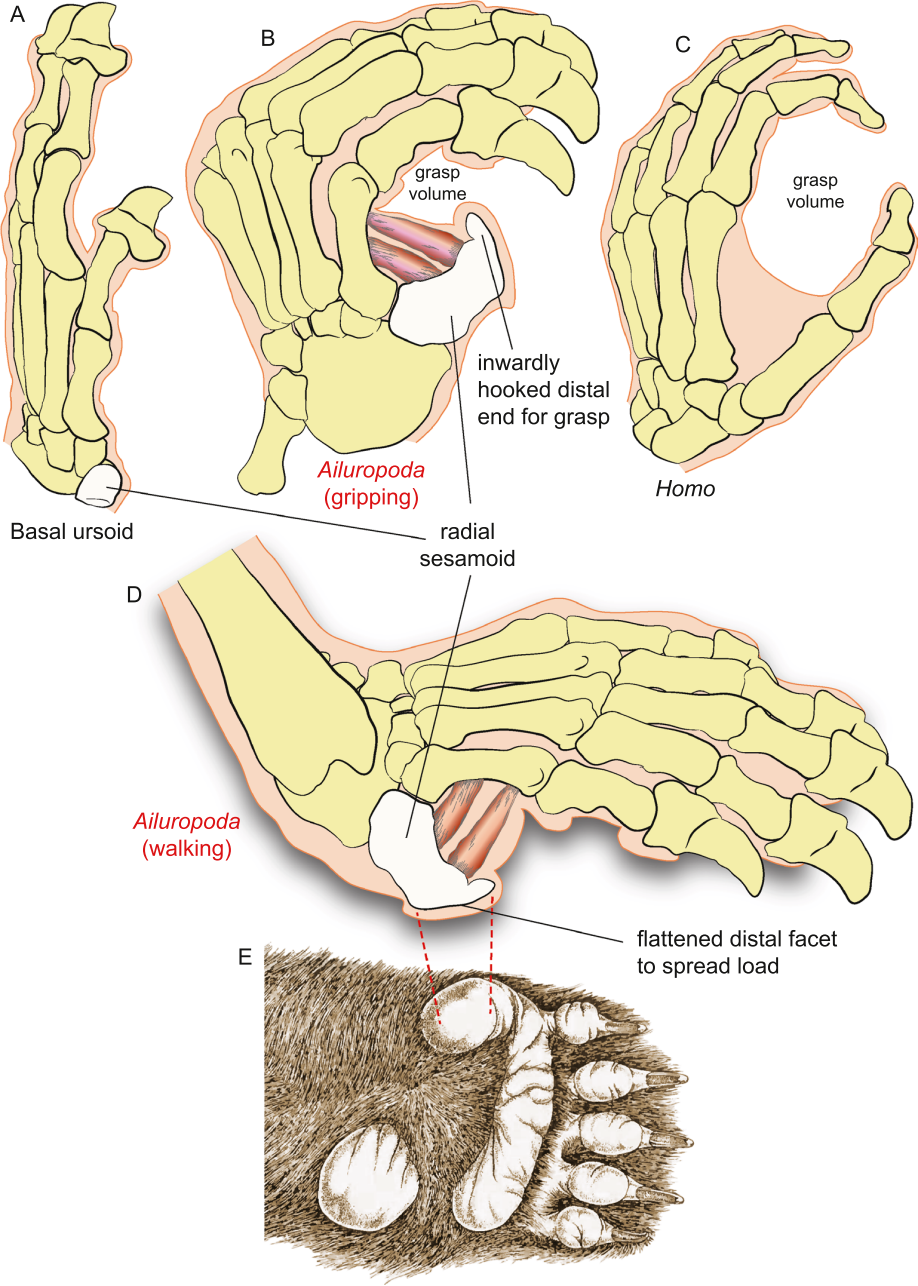
Comparison of the radial sesamoid in the basal ursoid, *Ailuropoda*, and *Homo* and the positioning of the radial sesamoid. Illustrations are of left hands. (**A**) A basal ursoid from the early Oligocene of North Dakota (USNM 637,259) showing the primitive condition of an unenlarged radial sesamoid; (**B**) grasping hand in extant *Ailuropoda*; (**C**) grasping hand of modern human; (**D**) walking hand of extant *Ailuropoda* in a plantigrade posture; (**E**) external ventral surface of the hand of *Ailuropoda* showing a fleshy, plantar pad that corresponds to the radial sesamoid (red dash lines), modified from Davis^8^. Muscles (dark red bundles) between the radial sesamoid and first metacarpal are *abductor pollicis brevis* and *opponens pollicis*, following Endo et al.^30^. Note the small distal hook and flat ventral surface of the radial sesamoid in extant *Ailuropoda*, which are derived features that function for better grasping (small hook) as well as walking (flattened palm surface) in contrast to the primitive conditions seen in *Ailurarctos* (Fig. 3).

Besides its comparatively large size, the radial sesamoid in *Ailurarctos* differs from that in modern *Ailuropoda* in other ways. A prominent tubercle arising from the inner edge of the articular facet for the scapholunar, presumably for the attachment of the *opponens pollicis* muscle^10,32^, is present in ZT-2015-0056 but is not seen in living *Ailuropoda*. Of more importance, a distinct hook in the distal end^10,32^, bending sharply inward toward the palm (Fig. 4C), and a correspondingly flattened external surface due to the thinning of the distal plate (Fig. 5C, D), both evident in extant *Ailuropoda,* are not present in *Ailurarctos* (see Dual Functions below for their functional significance). This flattened external, distal end corresponds to the accessory pad for the false thumb of the panda (Fig. 5E).

### Dual functions of false thumb

**Figure 6:**
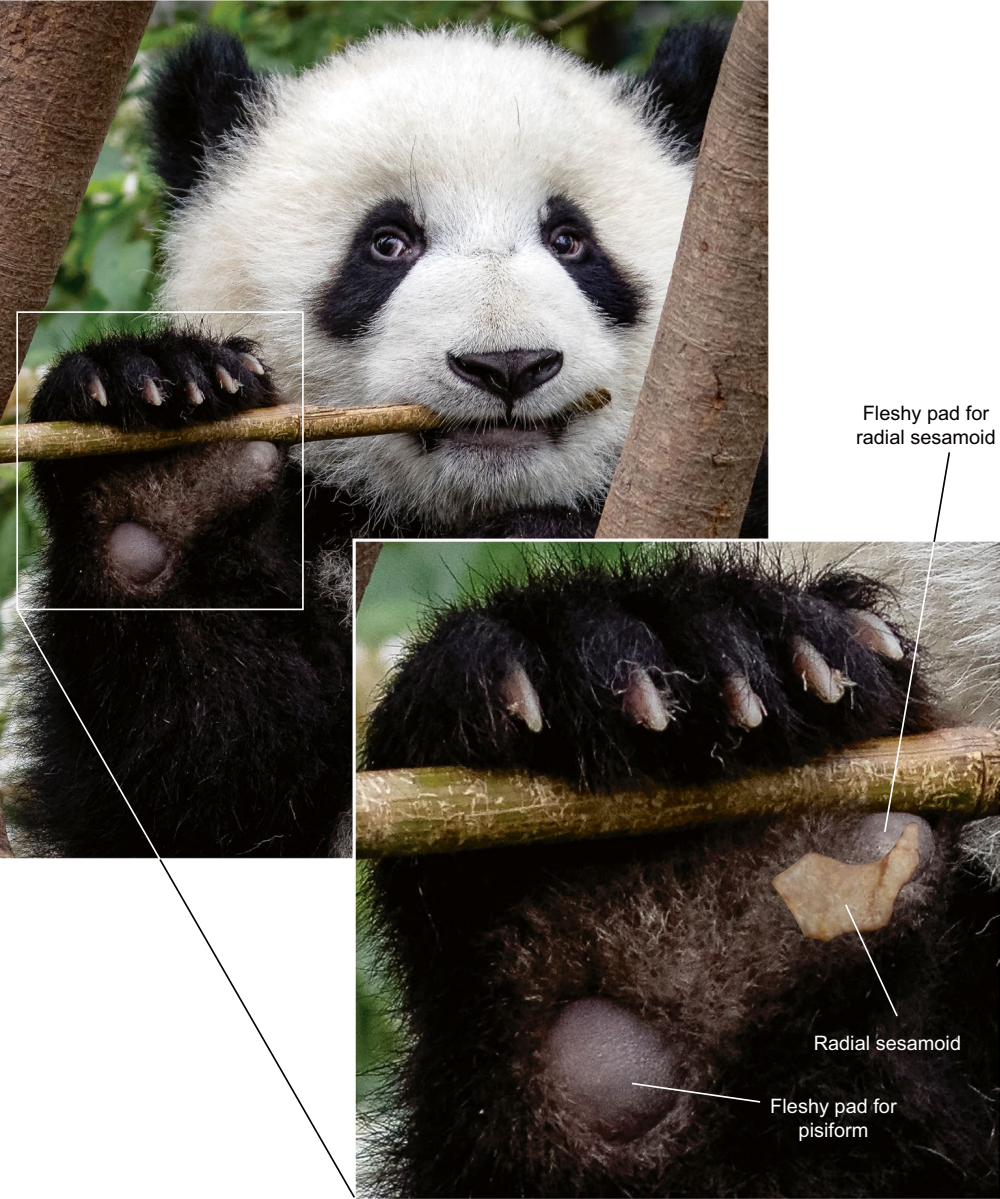
Giant panda gripping and chewing a thick, dried bamboo stem at Chengdu Research Base of Giant Panda Breeding on April 21, 2016. Inset: a semitransparent radial sesamoid bone is placed at its approximate position inside the fleshy pad (actual orientation of this bone may differ slightly from our placement). This photo also shows that the pisiform plays no role in bamboo grasping, *contra* Endo et al.^33^ Reproduction of photo by permission from Sharon Fisher.

Best developed in humans and their close relatives, precision grip by a true opposable thumb (capable of closure of the pollex to opposing fingers) requires not only flexibility of the joints but also complex interactions of flexor and extensor muscles. Endo et al.^33^ demonstrated that grasping in pandas is fundamentally different from that in humans. Instead of a human thumb that is capable of independent movements against other fingers, the panda’s radial sesamoid forms a functional complex in rigid articulation with the first metacarpal and scapholunar, which collectively rotate with other metacarpals. Once fully flexed, the radial sesamoid functional complex couples with the pisiform on the lateral side of the hand to function as a double stop against the pincer-like actions of the bending phalanges (but see Fig. 6, which shows only the radial sesamoid is used in the pincer action and the pisiform is not). Small muscles (such as *abductor pollicis brevis* and *opponens pollicis*) between the radial sesamoid and first metacarpal serve as a cushion for the bamboo stems grasped between the radial sesamoid and phalanges (Fig. 5). Such a passive system of gripping, far less effective than that of humans, nonetheless offers the panda the tightness of grip it needs for bamboo feeding. Furthermore, from an evolutionary point of view, such a simple passive mechanism of grasping can be functionally useful even with a slight initial enlargement of the radial sesamoid. Natural selection would be effective from the early stages of enlargement, i.e., even a small, protruding lump at the wrist can be a modest help in preventing bamboo from slipping off bent fingers.

**Figure 7:**
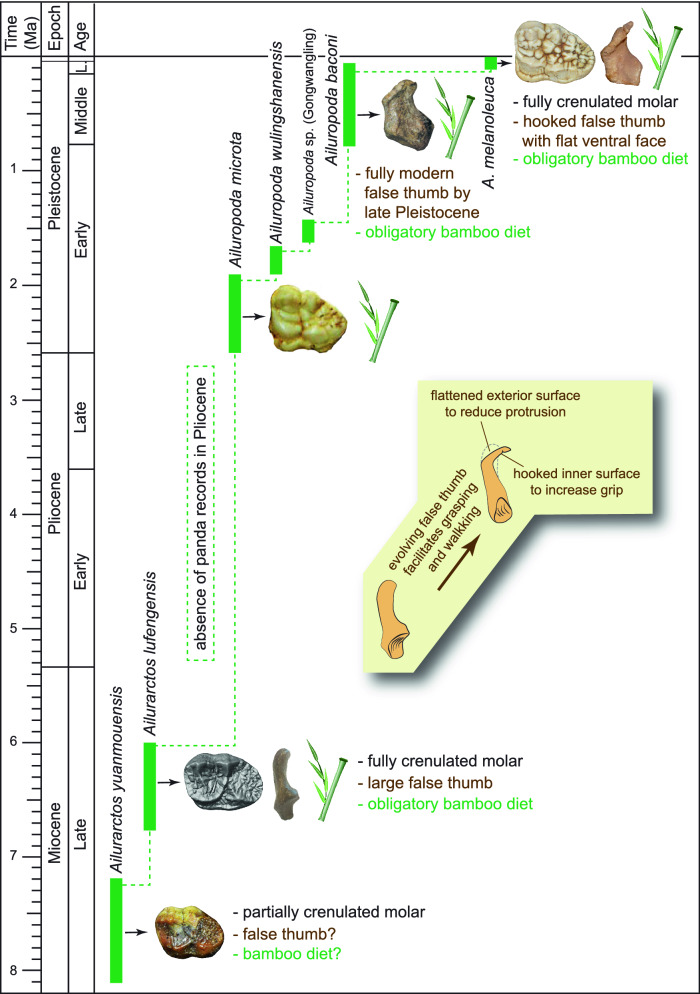
Phylogram of the giant panda showing the relevant steps discussed in the text and evolution of the false thumb. Pleistocene panda species ranges and chronospecies scheme follow Jin et al.^20^. The largest species, *Ailuropoda baconi*, was thought to be restricted to middle and late Pleistocene, with the modern species, *A. melanoleuca*, as Holocene^20^, although Sheng et al.^34^ recognized the initial divergence of the living lineage in the late Pleistocene. The false thumb from late Pleistocene Shuanghe Cave was adopted from Wang et al.^10^, which was referred to *A. melanoleuca*. A major hiatus exists in the Pliocene, likely reflecting poor Pliocene records in South China. Chronology of Lufeng and Yuanmou faunas is based on Dong and Qi^33^.

Radial sesamoids in living giant pandas have a rather abrupt, inward hook near the distal end (Figs. 4C, 5B, 7) as illustrated by Wood-Jones^30^ and Endo et al.^32^, and described by Davis^10^ and Wang et al.^12^. The function of this hook can be intuitively understood as a passive pincer in a single element grasping system, in contrast to that in humans with a two-segmented pollex in which the distal segment can be bent to facilitate grasping (Fig. 5). The lack of a distal hook in *Ailurarctos* indicates a two-step evolution, with an initial simple elongation in the false thumb followed by the subsequent appearance of a more refined distal hook (perhaps by late Pleistocene^12^), concomitant with a slight shortening of the tip.

The radial sesamoid in *Ailurarctos* exceeds that of its modern descendants, both in absolute and relative size (radial sesamoid index = 1.89 for *Ailurarctos* from Shuitangba; 0.92 for Shuanghe Cave fossil; 0.84–1.28 for living *Ailuropoda melanoleuca*) (Tables 1, 2). If a longer radial sesamoid alone was being selected, it would be expected that modern pandas would have increased the length of the radial sesamoid in the intervening six million years. Yet, modern pandas have a shorter radial sesamoid relative to their increased body size (as compared to their fossil ancestors), adding only a slight hook at the distal end. This raises the question of why the false thumb of pandas did not elongate further, as a longer digit would surely enhance capabilities for grasping thicker bundles of bamboo.

We propose that the lack of further elongation is the result of a functional compromise between the need for grasping larger bundles of bamboo and the weight-bearing function of the false thumb (Fig. 5). All ursids are fully plantigrade in their standing postures, i.e., the palm of the hand touches the ground while walking. A highly elongate radial sesamoid designed for bamboo manipulation would inevitably result in a conflict with walking long distances, thus compromising the radial sesamoid’s dual functions—its inner surface for grasping (Fig. 5B) and its outer surface for weight bearing (Fig. 5D). Due to its position in plantigrade posture, any further enlargement of the radial sesamoid would result in greater ventral protrusion and interference with walking. We view the flattened distal surface of the *Ailuropoda* radial sesamoid as a means to spread the load within the external accessory pad to cushion stride impact, an additional feature indicative of the dual functional demands of the radial sesamoid in both food procurement and locomotion. Living giant pandas thus balance these conflicting demands with a sharp bend inward on the distal end to form a hook and, at the same time, reducing external protrusion by flattening the external walking surface of the false thumb (Fig. 7).

As illustrated in the evolution of the false thumb (Fig. 5), more efficient consumption of bamboo cannot override requirements for weight-bearing while walking because the panda inherited a plantigrade posture. Potential alternatives to overcome such a constraint include a digitigrade posture (lifting the palm off the ground, freeing the wrist area from weight bearing), as in cursorial carnivorans (canids, felids, hyaenids), but this may not have been feasible for ursids given their evolutionary history of plantigrady in addition to arboreality. All digitigrade families evolved from a small, agile ancestor and efficient digitigrady evolved over millions of years, in contrast to ursids who were already large-bodied by the late Miocene and fully plantigrade^36^. Furthermore, pandas are partly arboreal, which is also facilitated by being plantigrade. Of the living ursids, only the giant pandas have a large fleshy pad (Fig. 6) to cushion the radial sesamoid^10^, signaling the importance of the weight-bearing function for this bone. While the panda solution may not be the most elegant, its functionality is evidenced by a long history of at least 6–7 million years (Fig. 1).

### Grasp volume and grip strength

The abundance of bamboo in the giant panda’s habitat makes daily foraging distance a very small component of the feeding strategy. Instead, eating fast and in large quantities appear to be of greater importance^37^. Perhaps the most demanding function of the false thumb is to maintain a tight grip on bamboo stems while the panda uses its teeth to tear and shred stems into bite size portions for consumption. The high strength of bamboo, especially the woody stems during the winter months, requires considerable grip strength by the hands to twist and jerk, countering the powerful biting and tearing by the jaws (see, for example, a panda cam at the San Diego Zoo: https://www.facebook.com/watch/live/?v=562351354170625&ref=watch_permalink). Therefore, it seems likely that a tight grip is more critical to panda’s feeding ability than the volume of their grasp.

The strength of the giant panda grip is dependent on the flexor muscles of the fingers, with the radial sesamoid acting as a passive stop against flexion of the fingers. Pandas are good climbers, especially for evading danger^37^, which necessitates powerful digital flexor muscles for the claws to penetrate into tree bark. Such musculature also serves well for gripping bamboo during feeding. Because of the functional constraints imposed on the length of the radial sesamoid noted above, pandas never evolved sufficiently long false thumbs to seize large bundles of bamboo, a task that, while desirable, is not critical for survival. It is instead the ability to grip tightly on bamboo stems to oppose strong twisting forces by the jaws that is essential and on which selection has acted. While the giant panda’s false thumb is not the most elegant or dexterous, the persistence of this distinctive morphology for the last six million years suggests that it has fulfilled an essential function for survival of the lineage.

### Bulk feeding as a tradeoff in low quality but year-round availability of bamboo

Besides having a false thumb, much else about the giant panda is also unusual and/or enigmatic. Pandas traded the high-protein, omnivorous diet of their ursid ancestors for bamboo, a woody grass of high fiber and low nutrition, but with year-round availability in South China and Southeast Asia. To make this tradeoff work, pandas eat prodigious quantities of bamboo, up to 45 kg/day (depending on the season), and spend ~ 15 h/day eating^37^. The panda’s short digestive tract, inherited from its carnivoran ancestors, is also poorly suited for extracting nutrients, absorbing less than 20% of digestible dry matter^38^. Furthermore, pandas lack the high-crowned teeth that most ungulate mammals possess for grinding tough plant fibers into a fine mush and consequently make minimal use of microbials to break down cellulose to extract the structural carbohydrates^11^. As a result, the panda’s gastrointestinal tract allows a rapid passage of digesta in less than 12 h^38^, too fast for fermentation when compared to fore- and hindgut fermenting mammalian herbivores, and necessitating an equally prodigious quantity of defecation, up to 100 times/day^39^.

This extreme bulk feeding strategy thus prioritizes dietary quantity over nutritional quality and nutrient extraction rate. This is made possible by the wide availability of bamboo, practically eliminating energy expenditure for foraging while maximizing the net rate of energy intake^40^. In modern panda habitats, bamboo (such as *Fargesia* and *Sinarundinaria*) are in plentiful, year-round supply, typically more than what pandas can consume (except during periodical bamboo flowering and die outs). With 99% of their food being bamboo and without major competitors for this abundant food resource, nor the need to avoid predators, pandas can thus reduce daily foraging range to within tens of meters of their resting dens, permitting a highly efficient foraging strategy of spending large portions of daily activities feeding and resting within small areas^37^.

Pandas usually feed while sitting, hooking bamboo stems toward the mouth using curved paws and while biting the leaves/stems, use grasping hands to jerk the stems up and down to help sever them^37^. The hands are also capable of a twisting action to tear off strips in the mouth, which requires a tight grip. It is difficult to imagine that the panda’s crude false thumb can be useful for conventional omnivorous purposes such as gathering of seeds, nuts, berries, or even low grasses, suggesting that the sole dietary purpose of an enlarged radial sesamoid is bamboo feeding, although a tree-climbing function for the false thumb has been suggested for ailurids^6,7^. If the dietary function is paramount, the panda’s false thumb must be a crucial adaptation for efficient bamboo procurement within this lineage. Once pandas committed to this bulk feeding strategy, the false thumb was an advantageous solution to the challenge of bamboo manipulation.

Despite the seeming inefficiency of its digestive system, the giant panda’s bulk feeding strategy permitted it to successfully expand to much of South China and Southeast Asia and become a prominent member of the Giant Panda-Stegodon fauna in the Pleistocene^41^ (Fig. 1). Deep inside the Chinese bamboo forests, giant pandas adopt a solitary, reclusive life of quiet herbivory, retreating from the more dominant position in the food chain of their distant relatives. In adopting a low-quality, year-round bamboo diet, pandas are also unable to store sufficient fat to hibernate, a crucial strategy for ursids to expand into high latitudes and to migrate across Beringia into North America^42,43^. The panda’s historic range is thus consistent with both the availability of bamboo and a warm climate without the need for hibernation (Fig. 1; note that a previous purported panda record from Zhoukoudian locality 1^44^, a jarring presence in a cold climate, has now been shown to be that of a cave bear^25^).

**Figure 8:**
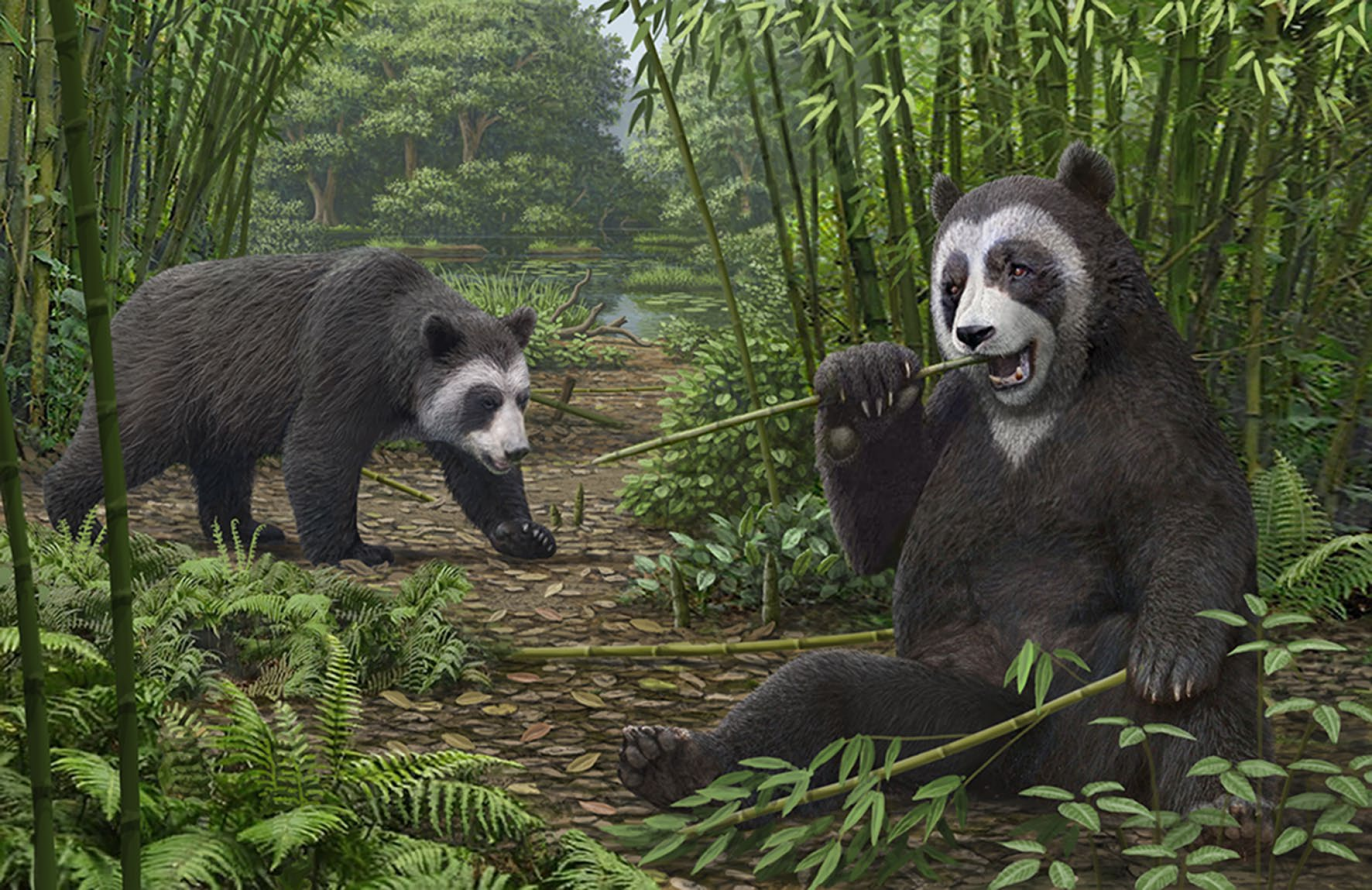
An artist reconstruction of *Ailurarctos* from Shuitangba. The grasping function of its false thumb (shown in the right individual) has reached to the level of modern pandas, whereas the radial sesamoid may have protruded slightly more than its modern counterpart during walking (seen in the left individual). Art by Mauricio Antón.

The panda’s transition from a broad, omnivorous diet to a highly specialized bamboo diet necessitated multiple changes in anatomy and physiology, as well as their genetics underpinning^45–47^. However, even after at least six million years of a bamboo diet, these transformations are still limited, mostly focused on food handling while the digestive system remains that of a carnivore^48^. The fact that there was no further elongation of the false thumb in the panda lineage after the late Miocene, suggests that an adequate grip for bamboo had been obtained, i.e., good enough for grasping a single stem or small bundle, and that further enlargement was inhibited by countervailing selection for weight-bearing and walking (Fig. 8). We caution, however, that the fossil record is too incomplete to allow a full understanding of this process and future discoveries will likely reveal unforeseen details.

Steven J. Gould’s^4^ insightful remarks still stand: “the panda’s true thumb is committed to another role, too specialized for a different function to become an opposable, manipulating digit. So the panda must use parts on hand and settle for an enlarged wrist bone and a somewhat clumsy, but quite workable, solution”. However, he would probably have been delighted to learn that the historic contingency of the panda’s false thumb requires that while being a better finger was favored by selection, it also had to bear the burden of considerable body weight.

## Supplementary Information

Supplementary Information.

## Data Availability

All data generated or analysed during this study are included in this published article and its supplementary information files.
