# Supplementary material for: Earliest giant panda false thumb suggests conflicting demands for locomotion and feeding
Source: Sci Rep. 2022 Jun 30;12:10538. doi: 10.1038/s41598-022-13402-y (PMC9246853; doi:10.1038/s41598-022-13402-y)
Supplement: Supplementary file 1 — Supplementary Information. [file 41598_2022_13402_MOESM1_ESM.docx]

Online Supplement

**Earliest giant panda false thumb suggests conflicting demands for locomotion and feeding**

Xiaoming Wang^1,2,*^, Denise F. Su^3^, Nina G. Jablonski^4^, Xueping Ji^5, 6^, Jay Kelley^3,7^, Lawrence J. Flynn^7^, Tao Deng^2^

^1^Department of Vertebrate Paleontology, Natural History Museum of Los Angeles County, 900 Exposition Blvd., Los Angeles, CA 90007, USA

^2^Key Laboratory of Vertebrate Evolution and Human Origins of Chinese Academy of Sciences, Institute of Vertebrate Paleontology and Paleoanthropology, Chinese Academy of Sciences, Beijing 100044, China

^3^Institute of Human Origins and School of Human Evolution and Social Change, Arizona State University, Tempe, Arizona, 85281, USA

^4^Department of Anthropology, Pennsylvania State University, University Park, Pennsylvania 16802, USA

^5^Kunming Natural history Museum of Zoology, Kunming Institute of Zoology, Chinese Academy of Sciences, Kunming 650201, China

^6^Yunnan Institute of Cultural Relics and Archaeology, 15-1, Chunmingli, Chunyuan Xiaoqu, Kunming, Yunnan 650118, China

^7^Department of Human Evolutionary Biology, Harvard University, Cambridge, Massachusetts 02138, USA

*Corresponding author. Email: xwang@nhm.org

# **A brief history of studies of radial sesamoids and alternative considerations**

The remarkably enlarged radial sesamoid in giant panda did not escape the notice of early naturalists. Lankester^1^ and Lydekker^2^ were the first to note this development, which they used as evidence to suggest a close relationship to the lesser (red) panda that also has an enlarged (but less extremely so than in the giant panda) radial sesamoid. Pocock^3^ was apparently the first to notice a peculiar, hook-like “plantar pad” that is associated with the radial sesamoid bone, and described the structure as a “supplementary digit”. Wood-Jones^4,5^ added additional observations and found much flexibility in this structure. In his authoritative monograph on giant panda anatomy, Davis^6^ noted the extraordinary development of radial sesamoid in the giant panda and made a comparison of radial and tibial sesamoids among *Ailuropoda*, *Tremarctos*, *Ursus*, *Ailurus*, and *Procyon*.

For wider studies, Ewer^7^ remarked that a radial sesamoid is present in all “canoid” carnivorans as well as in felids. In recent years, additional studies have expanded the occurrence to other fossil carnivorans. Abella et al.^8^ documented a large sample of radial sesamoids of *Indarctos arctoides* from the Spanish late Miocene Batallones-3 site, which was considered by some authors to be a distant member of the giant panda clade (subfamily Ailuropodinae)^9-11^. Salesa et al.^12,13^ described a false thumb in *Simocyon batalleri*, a distant relative of the red panda^14^. Salesa et al.^15^ further expanded their observations of a large radial sesamoid in tremarctine ursids as possibly a primitive condition for some ursids. Along similar themes, Antón et al.^16^ made observations of soft anatomy around the red panda radial sesamoid, as has also been investigated by Lankester ^1^ and Endo et al.^17,18^. A false thumb has even been described in a long-fingered lemur, the aye-aye (*Daubentonia madagascariensis*)^19^.

Based on the distal morphology, Abella et al.^8^ also hypothesized the presence of a cartilaginous tip in the radial sesamoid of the spectacled bear (*Indarctos arctoides*) that increases the functional length of the false thumb and adds insertion areas for the *abductor digiti I brevis* and *opponens digiti I* muscles, presumably increasing the leverage of these muscles. Such a cartilaginous tip has also been observed in living red panda^16^ as well as its distant relative, *Simocyon*^12^, but has not been seen in modern giant pandas, nor in the spectacled bear^15^. In the case of the red panda, *Ailurus fulgens*, the false thumb was hypothesized to initially serve a locomotor function for thin-branch climbing, later to be converted to bamboo manipulation^16^. It is perhaps also noteworthy that the red panda false thumb has a maximum length of 5.5 mm^16^, much too short (even after considering the cartilaginous tip) to be capable of grasping bamboo stems of significant thickness. The radial sesamoid of the spectacled bear, on the other hand, is substantially smaller than its giant panda counterpart, forming a blunt protuberance just slightly larger than other sesamoid bones in the carpus^15^. In the extinct *Indarctos*, an index of relative length of the radial sesamoid (0.401) is less than half of that for *Ailuropoda* (0.840), even smaller than those of *Ailurus* (0.451), *Simocyon* (0.506), and *Gulo* (0.510)^8^. In addition to the above differences in the size and shapes of radial sesamoids among arctoid carnivorans, the articulation facets are also different between ailuropodines and ailurines as noted by Endo et al.^17,18^, suggesting that grasping functions by the radial sesamoids in omnivorous carnivorans may have evolved independently.

# **Associated fauna, flora, age, and paleoenvironment**

Discovery of vertebrate fossils from Zhaotong Basin goes back to the 1960s^20^. Excavations at the Shuitangba site, a pit for brown-coal mining, was initiated by a Sino-American team of anthropologists, first reported by Su et al.^21^, and became well-known for its well-preserved hominoid. See Wang et al.^22^ for additional comments on the history of studies in Zhaotong Basin.

Jablonski et al.^23^ gave an overall account of the fauna from Shuitangba, which appears to be of the late Baodean Chinese Land Mammal Age (late Miocene) and is similar to faunas in Lufeng and Yuanmou basins^24^. A 14 m long Shuitangba fossil section and a nearby drill core can be magnetically correlated to the top of subchron C3An.1n through the base of C3An.2n^25^, with an age estimate of 6.03–6.73 Ma^26^. The hominoid fossils were recovered from a peaty clay layer calibrated to ~6.2 Ma^23,25,27^. Fossil vertebrates so far described from the Shuitangba site include: an extinct ape *Lufengpithecus* cf. *L. lufengensis*^25^, a muntjac *Muntiacus zhaotongensis*^28^, a tapir *Tapirus yunnanensis*^29^, proboscideans *Stegodon* and *Sinomastodon*^30^, small mammals such as the rodents “*Steneofiber*”, *Kowalskia*, and *Pliopetaurista*^23,25^ and the rabbit *Nesolagus*^31^, a giant otter *Siamogale melilutra*^22,32^, a pig *Propotamochoerus hyotherioides*^33^, a monkey *Mesopithecus pentelicus*^27^, and a rhinoceros *Acerorhinus lufengensis*^34^.

Pollen studies suggest a flora of evergreen broad-leaved forests with evergreen *Quercus* as the most dominant element, with expansion of grasses (including Poaceae) and decline of conifers at the hominoid-producing horizon^35^. A new species of aquatic plant, a fox nut (*Euryale yunnanensis*), was described from the Shuitangba site^36^, consistent with a shallow lake or swamp environment in a mildly warm and humid climate, also confirmed by sedimentological studies^37^. A seed of a new woody plant, *Zanthoxylum trachyspermum*, was published by Zhu et al.^38^. This genus also occurs in present-day Southwest China but is widespread in tropical, subtropical and temperate regions. A wider study of 14 seed taxa by Huang et al.^39^ suggests a stratified, mixed forest composed of trees, shrubs, lianas, herbs and grasses, near a lake occupied by various aquatic plants. Huang et al. also estimated a mean annual temperature (MAT) of 11.3–17.6°C and a mean annual precipitation (MAP) of 1,042–1,547 mm for the Shuitangba site. Most recently, Li et al.^40^ presented a paleoenvironmental study of the Zhaotong Basin based on chemical weathering indices and other proxies. They found that a gradual cooling trend prevails within generally warm and humid conditions in the region during the period of 8.8-6.2 Ma.

# **Additional descriptions of Shuitangba panda**

**Radial sesamoid**. We present a 3D model (Fig. S1) of the fossil radial sesamoid in both 3D PDF and PLY formats. These files were derived from photogrammetry software Agisoft Metashape.


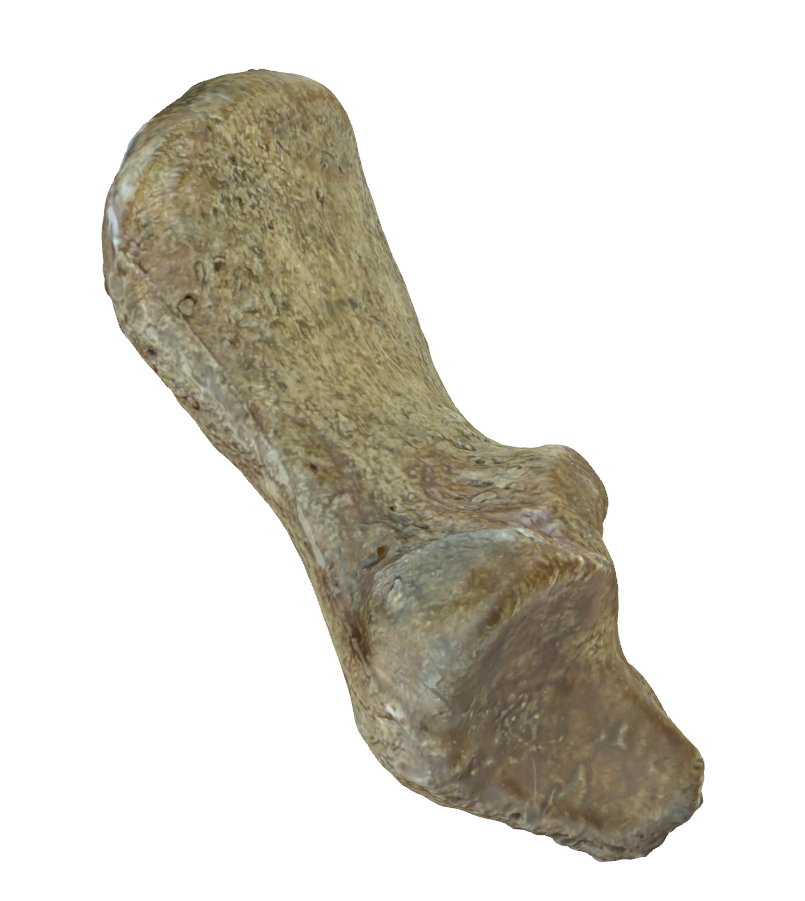


Figure S1. A screenshot of 3D model of left radial sesamoid, ZT-2015-0056, *Ailurarctos* cf. *A. lufengensis*. Original 3D photogrammetry files (in both PDF and PLY formats) can be downloaded from “Related file”.

**Canine**. A partial lower canine, ZT-2007-02-097 (Fig. S2), is missing much of the crown, only the basal part of the crown is preserved with the root. The most prominent feature of this tooth is its greatly thickened root, characteristic of ursid lower canines. The slightly anteriorly bowed root, suggesting a more recumbent crown, is consistent with it being a lower canine.


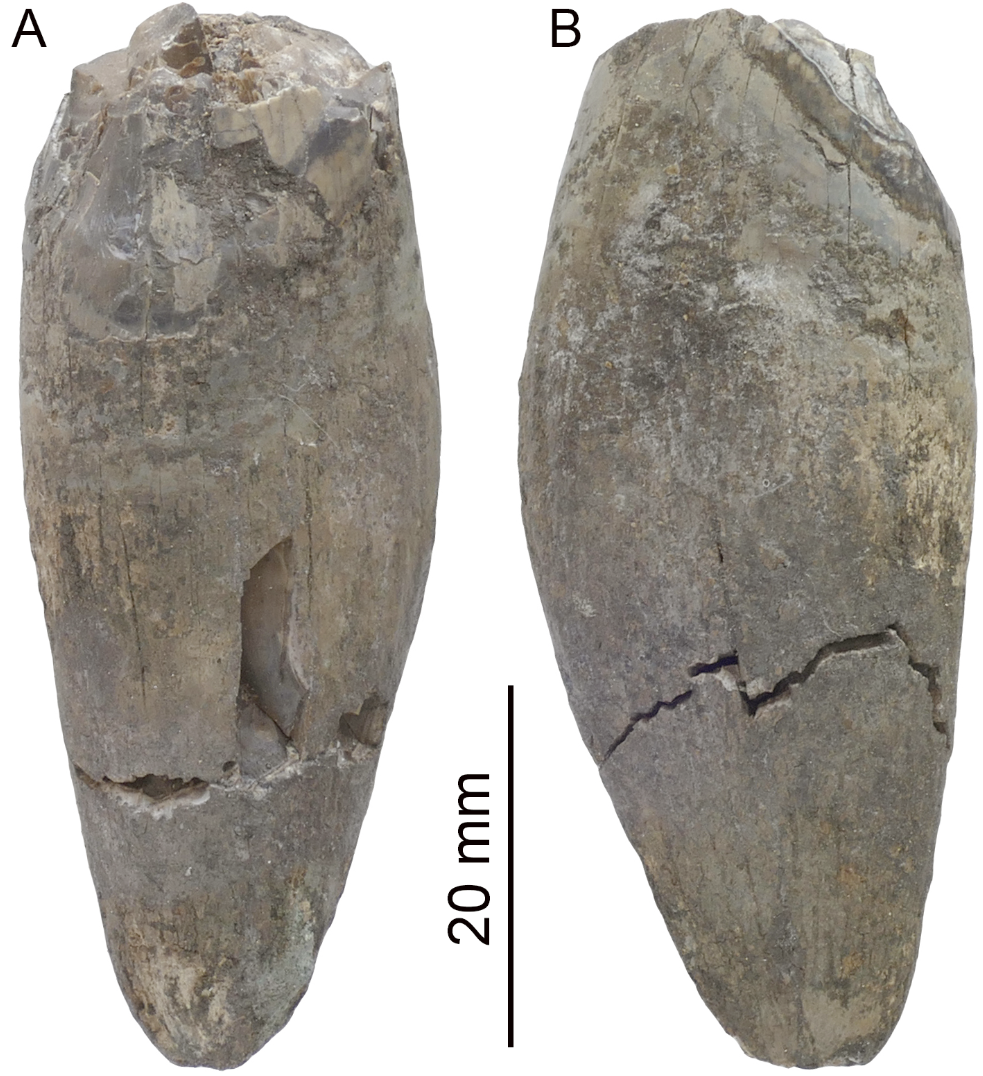


Figure S2. *Ailurarctos* cf. *A. lufengensis*, ZT-07-02-097. A, distal view and B, labial view of right (?) canine.

**Humerus**. ZT-2007-62-251 is a distal half of a left humerus (Fig. S3). The mediolateral diameter of the humeral trochlea, Hedml.TrL as defined by Figueirido et al. ^41^, is 41.35 mm and mediolateral diameter of the humeral epiphysis at mid shaft (equivalent to Hdml in Figueirido et al.^41^) is 20.63 mm. Using these measurements, we arrive at a body mass of ~51-70 kg, close to the lower end of modern female giant pandas (see Body Mass Estimate section below). In addition to being close to the size range of living giant pandas, the detailed morphology is also consistent with those of living pandas as described by Davis^6^ and Li and others^42^ (Fig. S4), such as a greatly expanded lateral supracondylar crest (epicondylar ridge in Davis^6^), a large medial epicondyle (incomplete), and a large entepicondylar foramen. The distal trochlea is relatively shallow and the olecranon fossa is not perforated, i.e., lack of a supratrochlear foramen. All of the above features suggest a lack of any cursorial adaptations but are consistent with a terrestrial, ambulatory ursid. Given that there is no other ursid in the Shuitangba fauna and that the size matches that of a giant panda, the assignment of this partial humerus to *Ailurarctos* is warranted. If this is correct, this is also the first record of a humerus in this taxon.


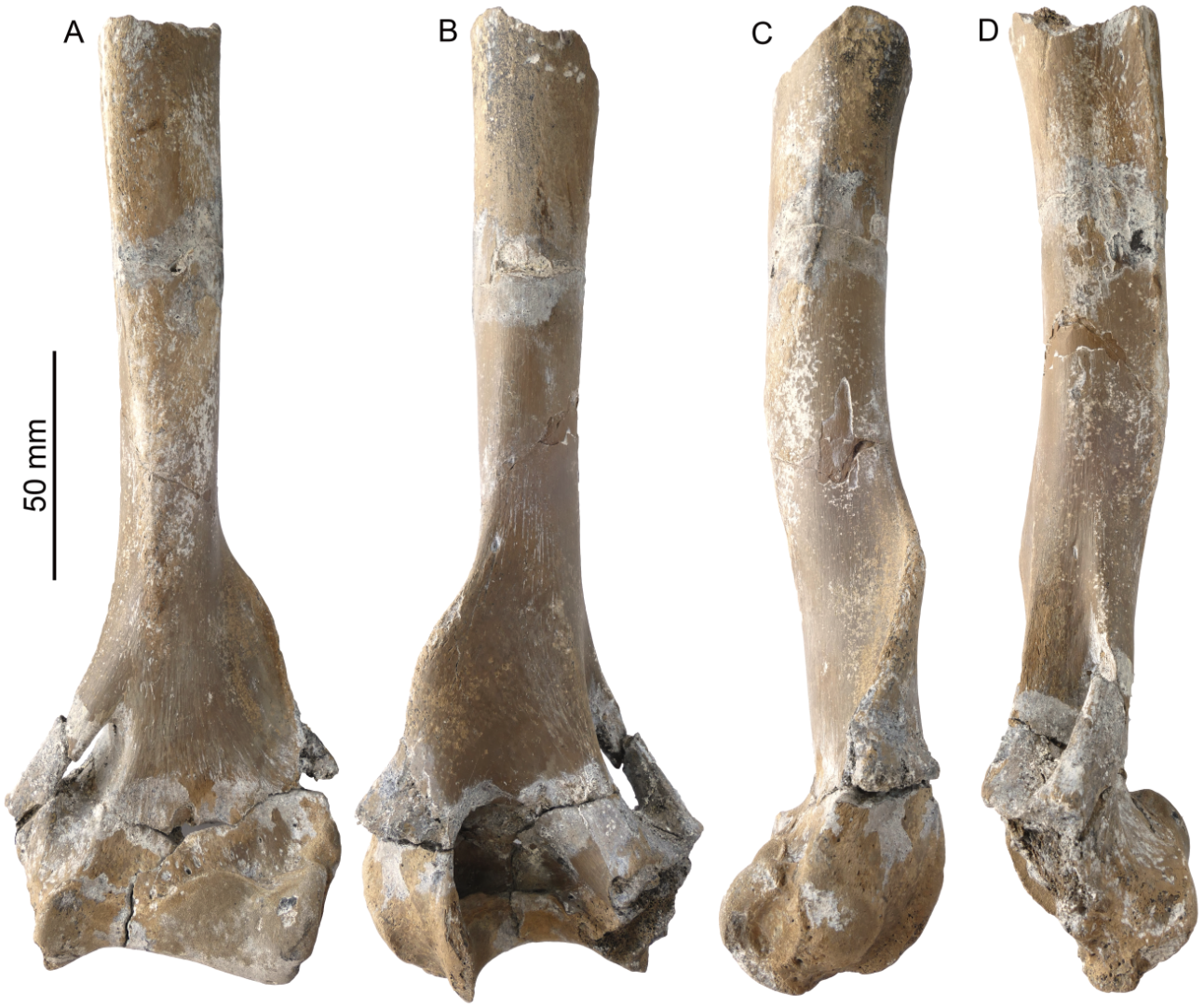


Figure S3. *Ailurarctos* cf. *A. lufengensis*, distal left humerus, ZT-2007-62-251. A, anterior, B, posterior, C, left lateral, and D, medial views.


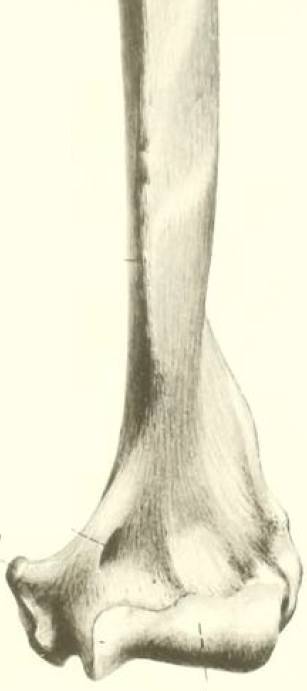

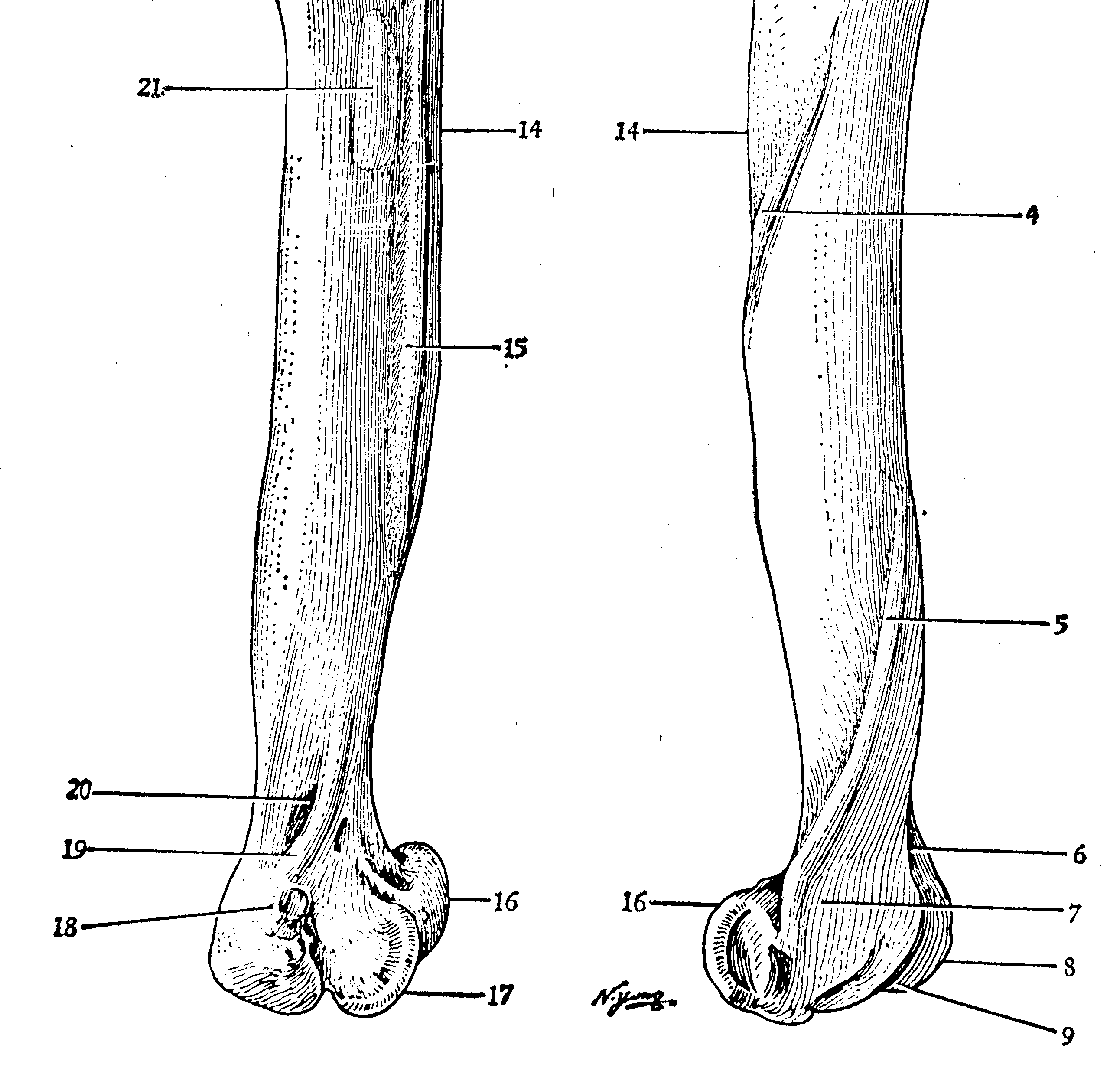


Figure S4. Illustrations of humerus of living *Ailuropoda melanoleuca*, for comparison with Shuitangba specimen (Fig. S3). Left: anterior view, adopted from Lankester^1^ (reversed from right); right: medial and lateral views, adopted from Li and others^42^; see the latter reference for anatomical structures indicated by the numbers.

The presence of an entepicondylar foramen on the humerus of the giant panda has been used to argue for an *Ailurus* (and procyonid) relationship by Lankester^1^ and Lydekker^2^, a notion no longer viable in modern understanding as the giant panda is firmly embedded within Ursidae in all recent genomic studies^43-45^. Although, as observed by Lankester and Lydekker, *Ursus* has lost the entepicondylar foramen, while more primitive tremarctine bears still have it (also confirmed by Davis^6^), suggesting a primitive presence of this structure in basal ursids. Lankester and Lydekker’s early study is apparently influential, as Pei^46^ made a similar argument, without citing Lankester and Lydekker, for the presence of a giant panda (“?*Ailuropus* sp.”) at the middle Pleistocene Locality 1 of Zhoukoudian. A partial distal humerus with a prominent entepicondylar foramen is the only available evidence for such a presence, although Pei^46^ cautioned that this may represent an “accidental recurrence of a rare feature in an ordinary bear”. Pei’s tentative determination, his caveat notwithstanding, also proves to be of far-reaching influence. Despite a total lack of dental materials, nearly all subsequent authors have duly allowed this extraordinary occurrence, far north of any plausible panda habitat, to be plotted as an outlier in their maps of historic distributions of the pandas^47-51^.

As remarked by Jiangzuo et al.^52^, the Zhoukoudian humerus is much too large for *Ailuropoda*. They estimated the width of the distal humerus condyle from the Zhoukoudian specimen as ~70 mm (85.5 mm by Pei’s own measurement), as compared to 44.6–49.5 mm in the living giant panda and 41.4 mm in *Ailurarctos* from Shuitangba, far exceeding the size of the humerus of any panda but comparable to those of cave bears, *Ursus deningeri*. As for the entepicondylar foramen, Erdbrink^53^ had offered an occasional example of its presence in cave bears. Jiangzuo et al.^52^ therefore concluded that the Zhoukoudian humerus is that of a cave bear. Jiangzuo et al.’s determination helps to eliminate one of the most spurious occurrences of giant panda fossil record by Pei^46^ that has been perpetuated by numerous subsequent authors^47-51,54-57^, despite its conspicuously jarring distribution far from Chinese bamboo forests (Fig. 1).

# **Notes on *Ailurarctos yuanmouensis***

Zong and Jiang^58^ mention a right M3 (probably a M2; ursids do not have a M3) and left and right m2s from Xiaohe Formation, Yuanmou Basin, Yunnan, and tentatively called these “*Ailurarctos* sp. nov.” They listed the occurrences at Baozidong (Leopard Cave) of IVPP loc. 8603 and Hudieliangzi (Butterfly Ridge) of IVPP loc. 8704. They remarked that the teeth are “essentially similar” to those of *A. lufengensis* but are smaller and simpler in structure. In his discussion about giant panda cranial and dental evolution, Huang^56^ first used the name “*Ailurarctos yuanmoensis*” but failed to provide a formal taxonomic description, which was thus a *nomen nudum*. *Ailurarctos yuanmouensis* was formally described by Zong^59^, a name adopted by later authors (e.g., Dong and Qi^24^).

# **Body mass estimate**

By adopting a regression equation by Figueirido et al.^41^,

Log10 (BM) = −1.874 (±0.096) + 1.400 (±0.337)Log10 (HedmlTrcl) + 1.061(±0.402) Log10(Hdml35%)

(The original formula has “Hdml35%” in the last item; the “35%” part is apparently an error that we eliminated). With HedmlTrcl=41.35 and Hdml=20.63 for ZT-2007-62-251, we arrive at a body mass estimate of 60.8 kg. We note that the above calculation is likely an underestimate of the body weight because of the incomplete nature of the distal humerus in ZT-2007-62-251, especially regarding the medial epicondyle as compared to complete one in living pandas (Figs. S3, S4). Our estimate thus should be viewed as a minimum body mass. The above formula has a prediction error of ±15.68% for ursids^41^. Therefore, *Ailurarctos* from Shuitangba has an estimated body weight of ~51-70 kg, which is on the lower end of modern female giant panda body weight^60^.

# **Additional remarks on bamboo feeding and panda distribution**

Pollen analysis of Shuitangba fossil site suggests a densely vegetated, moist and forested palaeoenvironment with a predominantly subtropical evergreen broad-leaf community mixed with a few temperate deciduous taxa and substantial aquatic plants^35^. Grasses (Poaceae) expanded at the hominoid-producing horizon (zone B), representing 9.8% of pollen counts. However, as is typical of fossil pollen analysis, bamboo pollens themselves are not separately treated within Poaceae.

It is perhaps telling that known giant pandas, including extinct species of *Ailurarctos* and *Ailuropoda*, are restricted to southern China, Burma^61^, Thailand^55^, and Vietnam^62^, mostly coinciding with the distribution of bamboo forests as panda’s stable food^51^ (green areas in Fig. 1). It thus seems likely that the availability of bamboo has been the main limiting factor of giant panda dispersal. A previously conspicuous outlier at the Zhoukoudian locality 1^46^ has now been shown to be false^52^ (see discussion under humerus above). By eliminating this false record, panda distribution is completely consistent with bamboo distribution.

Therefore, all known giant pandas must have been obligatory bamboo feeders, going back to their ancestors in the late Miocene, and this one-to-one relationship necessitates that they be tightly bound by the expansion and contraction of mid-latitude bamboo forests during the Pleistocene. Carbon isotope records from dental enamels of giant pandas show that they fed on bamboos (a C_3_ grass) throughout the Pleistocene^63^. Because they must eat a large quantity of low-quality bamboo, pandas must feed year-round. Therefore, unlike many other bears, especially those in cold climates of northern latitudes^64^, giant pandas cannot gain enough fat storage to hibernate for the winter. By comparison, red pandas, which are less exclusively dependent on bamboos^65^, were able to spread widely during the late Miocene and Pliocene, including to Europe and dispersal to North America^66^.

# **References**

1 Lankester, E. R. On the affinities of *Aeluropus melanoleucus*, A. Milne-Edwards. *Transactions of the Linnean Society of London, Series 2 Zoology* **8**, 163-165 (1901).

2 Lydekker, R. Detailed description of the skull and limb-bones. *Transactions of the Linnean Society of London, Series 2 Zoology* **8**, 166-171 (1901).

3 Pocock, R. I. Some external characters of the giant panda (*Ailuropoda melanoleuca*). *Proc. Zool. Soc. London* **1928**, 975-981 (1928).

4 Wood-Jones, F. The ‘thumb’ of the giant panda. *Nature* **143**, 246, doi:10.1038/143246b0 (1939).

5 Wood-Jones, F. The ‘thumb’ of the giant panda. *Nature* **143**, 157 (1939).

6 Davis, D. D. The giant panda, a morphological study of evolutionary mechanisms. *Fieldiana Zool. Mem.* **3**, 1-339 (1964).

7 Ewer, R. F. *The carnivores*. (Cornell University Press, 1973).

8 Abella, J. *et al.* Tracing the origin of the panda’s thumb. *Sci Nat* **102**, 1-13, doi:10.1007/s00114-015-1286-3 (2015).

9 Abella, J. *et al.* *Kretzoiarctos* gen. nov., the oldest member of the giant panda clade. *PLoS ONE* **7**, e48985, doi:10.1371/journal.pone.0048985 (2012).

10 Abella, J. *et al.* The last record of an ailuropod bear from the Iberian Peninsula. *geodiversitas* **41**, 797-809 (2019).

11 Qiu, Z.-x., Deng, T. & Wang, B.-y. A Late Miocene *Ursavus* skull from Guanghe, Gansu, China. *Vert. PalAsiat.* **52**, 265-302 (2014).

12 Salesa, M. J., Antón, M., Peigné, S. & Morales, J. Evidence of a false thumb in a fossil carnivore clarifies the evolution of pandas. *Proc. Nat. Acad. Sci.* **103**, 379-382 (2006).

13 Salesa, M. J., Antón, M., Peigné, S. & Morales, J. Functional anatomy and biomechanics of the postcranial skeleton of *Simocyon batalleri* (Viret, 1929) (Carnivora, Ailuridae) from the Late Miocene of Spain. *Zool. J. Linnean Soc.* **152**, 593-621 (2008).

14 Wang, X. New cranial material of *Simocyon* from China, and its implications for phylogenetic relationship to the red panda (*Ailurus*). *J. Vert. Paleontol.* **17**, 184-198 (1997).

15 Salesa, M. J., Siliceo, G., Antón, M., Montoya, P. & Morales, J. Anatomy of the "false thumb" of *Tremarctos ornatus* (Carnivora, Ursidae, Tremarctinae): phylogenetic and functional implications. *Est. Geol.* **62**, 389-394 (2006).

16 Antón, M., Salesa, M. J., Pastor, J. F., Peigné, S. & Morales, J. Implications of the functional anatomy of the hand and forearm of *Ailurus fulgens* (Carnivora, Ailuridae) for the evolution of the ‘false-thumb’ in pandas. *Journal of Anatomy* **209**, 757-764 (2006).

17 Endo, H., Sasaki, M., Kogiku, H., Yamamoto, M. & Arishima, K. Radial sesamoid bone as a part of the manipulation system in the lesser panda (*Ailurus fulgens*). *Annals of Anatomy* **183**, 181-184 (2001).

18 Endo, H. *et al.* Three-dimensional analysis of the manipulation system in the lesser panda. *Mammal Study* **32**, 99-103 (2007).

19 Hartstone-Rose, A., Dickinson, E., Boettcher, M. L. & Herrel, A. A primate with a Panda's thumb: The anatomy of the pseudothumb of *Daubentonia madagascariensis*. *American Journal of Physical Anthropology* **171**, 8-16, doi:10.1002/ajpa.23936 (2020).

20 Chow, M.-c. & Zhai, R.-j. Early Pleistocene mammals of Chaotung, Yunnan, with notes on some Chinese stegodonts. *Vert. PalAsiat.* **6**, 138-147 (1962).

21 Su, D. F., Ji, X.-p., Kelley, J. & Jablonski, N. G. A new paleontological site in the Zhaotong Basin of Yunnan Province, China. *American Journal of Physical Anthropology* **138(S48)**, 360, doi:doi:10.1002/ajpa.21030 (2009).

22 Wang, X. *et al.* A new otter of giant size, *Siamogale melilutra* sp. nov. (Lutrinae: Mustelidae: Carnivora), from the latest Miocene Shuitangba site in north-eastern Yunnan, south-western China, and a total-evidence phylogeny of lutrines. *Journal of Systematic Palaeontology* **15**, 1-27, doi:10.1080/14772019.2016.1267666 (2017).

23 Jablonski, N. G. *et al.* The site of Shuitangba (Yunnan, China) preserves a unique, terminal Miocene fauna. *J. Vert. Paleontol.* **34**, 1251-1257, doi:10.1080/02724634.2014.843540 (2014).

24 Dong, W. & Qi, G.-q. in *Fossil Mammals of Asia: Neogene Biostratigraphy and Chronology* (eds Xiaoming Wang, Lawrence J. Flynn, & Mikael Fortelius) 293-313 (Columbia University Press, 2013).

25 Ji, X.-P. *et al.* Juvenile hominoid cranium from the terminal Miocene of Yunnan, China. *Chinese Sci. Bull.*, 1-9, doi:10.1007/s11434-013-6021-x (2013).

26 Hilgen, F. J. *et al.* in *The Geologic Time Scale 2012, Volume 2* (eds Felix M. Gradstein, James G. Ogg, Mark D. Schmitz, & Gabi M. Ogg) 923-978 (Elsevier, 2012).

27 Jablonski, N. G. *et al.* *Mesopithecus pentelicus* from Zhaotong, China, the easternmost representative of a widespread Miocene cercopithecoid species. *J. Human Evol.* **146**, 102851, doi:<https://doi.org/10.1016/j.jhevol.2020.102851> (2020).

28 Dong, W., Ji, X.-P., Jablonski, N. G., Su, D. F. & Li, W.-Q. New materials of the Late Miocene *Muntiacus* from Zhaotong hominoid site in southern China. *Vert. PalAsiat.* **52**, 316-327 (2014).

29 Ji, X.-p. *et al.* *Tapirus yunnanensis* from Shuitangba, a terminal Miocene hominoid site in Zhaotong, Yunnan Province of China. *Vert. PalAsiat.* **53**, 177-192 (2015).

30 Wang, S.-q. *et al.* The oldest cranium of *Sinomastodon* (Proboscidea, Gomphotheriidae), discovered in the uppermost Miocene of southwestern China: Implications for the origin and migration of this taxon. *J. Mamm. Evol.*, 1-19, doi:10.1007/s10914-015-9311-z (2015).

31 Flynn, L. J. *et al.* Late Miocene fossil calibration from Yunnan Province for the striped rabbit *Nesolagus*. *Vert. PalAsiat.* **57**, 214-224 (2019).

32 Tseng, Z. J., Su, D. F., Wang, X., White, S. C. & Ji, X. Feeding capability in the extinct giant *Siamogale melilutra* and comparative mandibular biomechanics of living Lutrinae. *Scientific Reports* **7**, 15225, doi:10.1038/s41598-017-15391-9 (2017).

33 Hou, S. *et al.* New fossil suid specimens from the terminal Miocene hominoid locality of Shuitangba, Zhaotong, Yunnan Province, China. *J. Mamm. Evol.* **26**, 557-571, doi:10.1007/s10914-018-9431-3 (2019).

34 Lu, X. *et al.* Palaeoenvironment examination of the terminal Miocene hominoid locality of the Zhaotong Basin, southwestern China, based on the rhinocerotid remains. *Hist. Biol.*, 1-9, doi:10.1080/08912963.2017.1360294 (2017).

35 Chang, L. *et al.* Pollen evidence of the palaeoenvironments of *Lufengpithecus lufengensis* in the Zhaotong Basin, southeastern margin of the Tibetan Plateau. *Palaeogeog. Palaeoclim. Palaeoecol.* **435**, 95-104, doi:<http://dx.doi.org/10.1016/j.palaeo.2015.06.007> (2015).

36 Huang, Y.-j. *et al.* Fossil seeds of *Euryale* (Nymphaeaceae) indicate a lake or swamp environment in the late Miocene Zhaotong Basin of southwestern China. *Sci. Bull.* **60**, 1768-1777, doi:10.1007/s11434-015-0870-4 (2015).

37 Zhang, C. *et al.* Clay mineralogy indicates a mildly warm and humid living environment for the Miocene hominoid from the Zhaotong Basin, Yunnan, China. *Scientific Reports* **6**, 20012, doi:10.1038/srep20012

<http://www.nature.com/articles/srep20012#supplementary-information> (2016).

38 Zhu, H., Huang, Y.-J., Ji, X.-P., Su, T. & Zhou, Z.-K. Continuous existence of *Zanthoxylum* (Rutaceae) in Southwest China since the Miocene. *Quat. Int.* **392**, 224-232, doi:<https://doi.org/10.1016/j.quaint.2015.05.020> (2016).

39 Huang, Y.-J. *et al.* Habitat, climate and potential plant food resources for the late Miocene Shuitangba hominoid in Southwest China: Insights from carpological remains. *Palaeogeog. Palaeoclim. Palaeoecol.* **470**, 63-71, doi:<https://doi.org/10.1016/j.palaeo.2017.01.014> (2017).

40 Li, P. *et al.* Late Miocene climate cooling contributed to the disappearance of hominoids in Yunnan region, southwestern China. *Geophys. Res. Letters* **47**, e2020GL087741, doi:10.1029/2020gl087741 (2020).

41 Figueirido, B., Pérez-Claros, J. A., Hunt, R. M., Jr. & Palmqvist, P. Body mass estimation in amphicyonid carnivoran mammals: A multiple regression approach from the skull and skeleton. *Acta Palaeontol. Polonica* **56**, 225-246 (2011).

42 Li, Y.-w. & others. *Morphology of the Giant Panda, Systematic Anatomy and Organ-Histology (in Chinese)*. (Science Press, 1986).

43 Nakagome, S., Pecon-Slattery, J. & Masuda, R. Unequal rates of Y chromosome gene divergence during speciation of the family Ursidae. *Mol. Biol. Evol.* **25**, 1344-1356, doi:10.1093/molbev/msn086 (2008).

44 Kutschera, V. E. *et al.* Bears in a forest of gene trees: Phylogenetic inference is complicated by incomplete lineage sorting and gene flow. *Mol. Biol. Evol.* **31**, 2004–2017, doi:10.1093/molbev/msu186 (2014).

45 Kumar, V. *et al.* The evolutionary history of bears is characterized by gene flow across species. *Scientific Reports* **7**, 46487, doi:10.1038/srep46487

<https://www.nature.com/articles/srep46487#supplementary-information> (2017).

46 Pei, W.-c. On the Carnivora from Locality 1 of Choukoutien. *Palaeontol. Sinica Ser. C* **8**, 1-216 (1934).

47 Han, H. *et al.* Diet evolution and habitat contraction of giant pandas via stable isotope analysis. *Current Biology* **29**, 664-669.e662, doi:<https://doi.org/10.1016/j.cub.2018.12.051> (2019).

48 Loucks, C. J. *et al.* Giant pandas in a changing landscape. *Science* **294**, 1465-1465, doi:10.1126/science.1064710 (2001).

49 Jablonski, N. G. *et al.* Remains of Holocene giant pandas from Jiangdong Mountain (Yunnan, China) and their relevance to the evolution of quaternary environments in south-western China. *Hist. Biol.* **24**, 527-536, doi:10.1080/08912963.2011.640400 (2012).

50 Sheng, G.-L. *et al.* Ancient DNA from giant panda (*Ailuropoda melanoleuca*) of south-western China reveals genetic diversity loss during the Holocene. *Genes*, 16, doi:10.3390/genes9040198 (2018).

51 Chen, Y.-p., Ellison, A. M. & Lu, Y.-l. Establish a special conservation zone for the captive giant panda. *Ecosystem Health and Sustainability* **4**, 29-33, doi:10.1080/20964129.2018.1455990 (2018).

52 Jiangzuo, Q.-g. *et al.* Presence of the Middle Pleistocene cave bears in China confirmed – Evidence from Zhoukoudian area. *Quat. Sci. Rev.* **199**, 1-17, doi:<https://doi.org/10.1016/j.quascirev.2018.09.012> (2018).

53 Erdbrink, D. P. *A review of fossil and recent bears of the Old World with remarks on their phylogeny based upon their dentition. Part I*. (Drukkerij Jan de Lange, 1953).

54 Wang, T.-k. On the taxonomic status of species, geological distribution and evolutionary history of *Ailuropoda*. *Acta Zool. Sinica* **20**, 191-201 (1974).

55 Tougard, C., Chaimanee, Y., Suteethorn, V., Triamwichanon, S. & Jaeger, J.-J. Extension of the geographic distribution of the giant panda (*Ailuropoda*) and search for the reasons for its pregressive disappearance in Southeast Asia during the latest Middle Pleistocene. *Comptes Rendus de l'Académie des Sciences Paris, Series II a* **323**, 973-979 (1996).

56 Huang, W.-b. The skull, mandible and dentition of giant pandas (*Ailuropoda*): morphological characters and their evolutionary implications. *Vert. PalAsiat.* **31**, 191-207 (1993).

57 Li, T., Lai, X.-L., Wang, W. & Zhou, X.-g. Taxonomy and evolution of giant panda. *Geological Science and Technology Information* **23**, 40-46 (2004).

58 Zong, G.-f. & Jiang, C. A preliminary observation on Carnivora from the Neogene locality of Yuanmou, Yunnan. *Vert. PalAsiat.* **29**, 136-142 (1991).

59 Zong, G.-f. in *Yuanmou Hominoid Fauna* (ed Z.-q. He) 69-89 (Yunnan Science and Technology Press, 1997).

60 Chorn, J. & Hoffmann, R. S. *Ailuropoda melanoleuca*. *Mamm. Species* **110**, 1-6 (1978).

61 Woodward, A. S. On the skull of an extinct mammal related to *Aeluropus* from a cave in the Ruby Mines at Mogok, Burma. *Proc. Zool. Soc. London* **1915**, 425-428 (1915).

62 Long, V. T., de Vos, J. & Ciochon, R. The fossil mammal fauna of the Lang Trang caves, Vietnam, compared with Southeast Asian fossil and recent mammal faunas: the geographical implication. *Bulletin of the Indo-Pacific Prehistory Association* **14**, 38-56 (1996).

63 Stacklyn, S. *et al.* Carbon and oxygen isotopic evidence for diets, environments and niche differentiation of early Pleistocene pandas and associated mammals in South China. *Palaeogeog. Palaeoclim. Palaeoecol.* **468**, 351-361, doi:<http://dx.doi.org/10.1016/j.palaeo.2016.12.015> (2017).

64 Wang, X., Rybczynski, N., Harington, C. R., White, S. C. & Tedford, R. H. A basal ursine bear (*Protarctos abstrusus*) from the Pliocene High Arctic reveals Eurasian affinities and a diet rich in fermentable sugars. *Scientific Reports* **7**, 17722, doi:10.1038/s41598-017-17657-8 (2017).

65 Roberts, M. S. & Gittleman, J. L. *Ailurus fulgens*. *Mamm. Species* **222**, 1-8 (1984).

66 Wallace, S. C. & Wang, X. Two new carnivores from an unusual late Tertiary forest biota in eastern North America. *Nature* **431**, 556-559 (2004).
